# Cerium oxide nanoparticles with antioxidant capabilities and gadolinium integration for MRI contrast enhancement

**DOI:** 10.1038/s41598-018-25390-z

**Published:** 2018-05-03

**Authors:** Peter Eriksson, Alexey A. Tal, Andreas Skallberg, Caroline Brommesson, Zhangjun Hu, Robert D. Boyd, Weine Olovsson, Neal Fairley, Igor A. Abrikosov, Xuanjun Zhang, Kajsa Uvdal

**Affiliations:** 1 Division of Molecular Surface Physics and Nanoscience, Department of Physics, Chemistry and Biology (IFM), Linköping University, SE-581 83 Linköping, Sweden; 2 Division of Theoretical Physics, Department of Physics, Chemistry and Biology (IFM), Linköping University, SE-581 83 Linköping, Sweden; 3 Plasma Coatings Physics, Department of Physics, Chemistry and Biology (IFM), Linköping University, SE-581 83 Linköping, Sweden; 4 Casa Software Ltd, Bay House, 5 Grosvenor Terrace, Teignmouth, TQ14 8NE United Kingdom; 5 Materials Modeling and Development Laboratory, National University of Science and Technology “MISIS”, 119049 Moscow, Russia; 6 Faculty of Health Sciences, University of Macau, Macau, SAR China

## Abstract

The chelating gadolinium-complex is routinely used as magnetic resonance imaging (MRI) -contrast enhancer. However, several safety issues have recently been reported by FDA and PRAC. There is an urgent need for the next generation of safer MRI-contrast enhancers, with improved local contrast and targeting capabilities. Cerium oxide nanoparticles (CeNPs) are designed with fractions of up to 50% gadolinium to utilize the superior MRI-contrast properties of gadolinium. CeNPs are well-tolerated *in vivo* and have redox properties making them suitable for biomedical applications, for example scavenging purposes on the tissue- and cellular level and during tumor treatment to reduce *in vivo* inflammatory processes. Our near edge X-ray absorption fine structure (NEXAFS) studies show that implementation of gadolinium changes the initial co-existence of oxidation states Ce^3+^ and Ce^4+^ of cerium, thereby affecting the scavenging properties of the nanoparticles. Based on a*b initio* electronic structure calculations, we describe the most prominent spectral features for the respective oxidation states. The as-prepared gadolinium-implemented CeNPs are 3–5 nm in size, have r_1_-relaxivities between 7–13 mM^−1^ s^−1^ and show clear antioxidative properties, all of which means they are promising theranostic agents for use in future biomedical applications.

## Introduction

Today there is an urgent need to obtain nanoprobes and biomarkers with capability to identify (find and report) the hallmarks for, and subsequently treat at the tissue and cell level, specific diseases. These are termed theranostic agents with combined diagnostic and therapeutic properties. Theranostic nanoprobes may be an important tool for disease diagnosis by obtaining improved visualization and monitor image-guided therapy^1,2^. The standard clinical approach today is focused on developing general medicines for well characterized diseases^3^. This kind of treatment is not optimal when it comes to more complex diseases with heterogeneous expressions such as cancer tumors^4,5^, requiring tailor-made personalized treatment such as nanoparticle-based theranostic agents^6^.

Nanoparticles possess several important physiochemical properties which makes them suitable for biomedical applications including optimal size, large surface area to mass ratio, high reactivity and the ability to modify their biomedical parameters such as blood circulation time, diffusivity and immunogenicity^7^. Theranostic nanoparticles can be constructed in many different ways^8–11^ in order to obtain the desired properties, for example conjugating therapeutic agents to imaging nanoparticles, imaging agents to therapeutic nanoparticles and engineering unique nanoparticles possessing both therapeutic and diagnostic abilities^12^. In this study, cerium oxide nanoparticles (CeNPs) with varying gadolinium (Gd) content have been designed to obtain nanoparticles possessing intrinsic theranostic properties suitable for biomedical applications.

Cerium (Ce) and Gd possess shielded 4f-electrons, which are responsible for the fascinating properties of the rare earth elements. Ce has electronic configuration [Xe]4 f^2^6s^2^ and has two common oxidation states Ce^3+^ and Ce^4+^ ^13^. In cerium dioxide form it adopts cubic fluorite crystal structure and displays a range of interesting physical and chemical properties such as strong UV-absorption^14^, high optical transparency in the visible region^14^, high refractive index^13^, interesting redox properties^15^, high oxygen storage- and releasing capacity^16^. This is why it is a highly promising material for a wide range of applications as for example electrolytes in solid oxide fuel cells^17^, UV-filter^18^, fuel additives^19^, solar cells^20^, catalytic material and others^21^.

In the biomedical field, CeNPs have attracted special interest for their regenerative, multi-enzymatic scavenging of reactive oxygen species (ROS)^22–24^. CeNPs’ unique antioxidant/catalytic properties stem from 1) the coexistence of oxidation states 3+ (Ce^3+^) and 4+ (Ce^4+^), 2) the reversible switching between these states and 3) the low reduction potential of ~1.52 V^23^. Cerium dioxide as a bulk crystal mainly consists of Ce^4+^, but reduction in size to nano-dimensions significantly enhances the relative amount of Ce^3+^ ^25^ resulting in higher catalytic effects which are comparable to the capabilities of biological antioxidants^26^ and highly relevant for biological processes.

Production of ROS is a key mechanism in the immune response following infection. However, excessive and misdirected production of ROS can cause inflammatory disorders such as cancer^27^, neurodegenerative disease, diabetes and cardiovascular diseases^28^. The scavenging ability of cerium oxide is known to be pH-dependent, meaning that in a neutral environment CeNPs have multi-enzymatic abilities while in acidic environments, which may be the case in cancerous tissue, the CeNPs instead show pro-oxidant abilities^23^. CeNPs capability to switch in anti-/pro-oxidant properties can be utilized for sensitizing cancer cells for radiation therapy while protecting normal cells^29,30^.

The Ce^3+^-proportion and the biocatalytic properties of CeNPs can be further enhanced upon by the implementation of trivalent rare-earth elements^22^, including Gd which possesses seven unpaired 4f-electrons with parallel spins, a large magnetic moment and a slow electronic relaxation rate all of which are optimal properties for MRI contrast enhancement^31^. Gd is the most prominently used material for MRI contrast enhancement in clinic^32^. Gd has an ionic radius of 1.053 Å which is between the ionic radius of Ce^4+^ (0.970 Å) and Ce^3+^ (1.143 Å) and is therefore straight forward to incorporate into the CeNPs’ matrix and will actually increase the Ce^3+^ state in CeNPs. Babu *et al*. demonstrated experimentally that the fraction concentration of Gd can be 40% and could be even higher according to theoretical *ab initio* calculations^33^.

There are toxicity concerns associated with the use of Gd based contrast agents due to the high toxicity of free Gd-ions i.e. those not bound into a crystal structure or incorporated into an organic complex^34,35^. A strategy to reduce the dosage of Gd is needed to improve the contrast enhancement ability (relaxivity) through the construction of Gd containing nanoparticles^36^, stabilize the particles with biocompatible capping layer and then conjugate with the targeting biomolecules^35^. Previously we have developed several kinds of Gd based nanoparticles for MRI contrast enhancement^37–39^, which were well-tolerated by Ba/F3 cell lines^40^ and also used capping strategies in order to increase gadolinium oxide nanoparticles´ biocompatibility^41,42^. Additionally, reduction of free Gd exposure has been taken into consideration by its implementation into the crystal structure of Ce.

**Figure 1:**
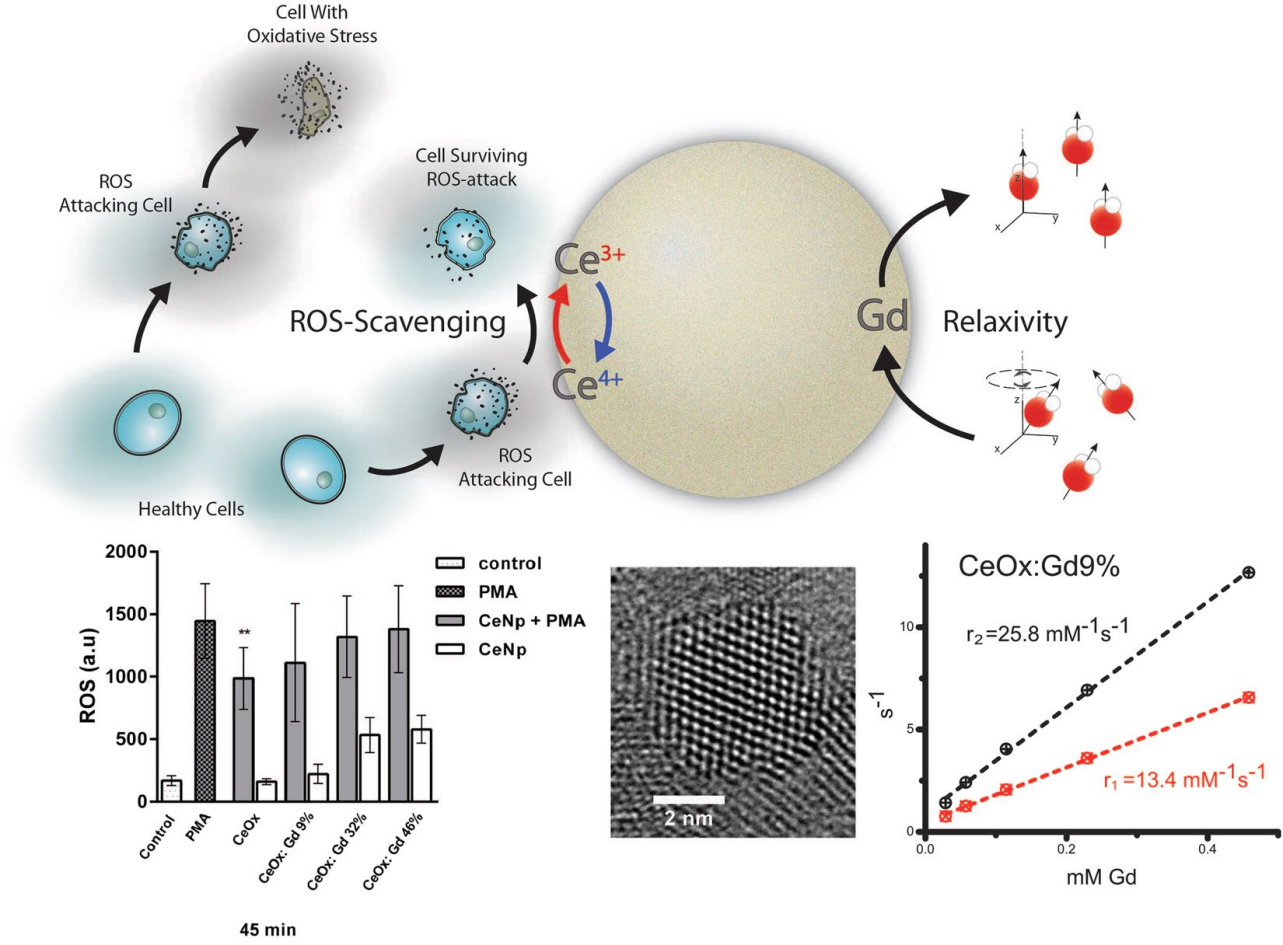
Top: schematic illustration of a gadolinium-integrated cerium oxide nanoparticle possessing antioxidant and MRI contrast enhancing properties. Bottom: results from (left to right) antioxidant assay, HRTEM and relaxivity measurement.

In this study, the synthesized Gd containing CeNPs have been designed and carefully characterized using dynamic light scattering (DLS), Zeta potential, X-ray diffraction (XRD), high- resolution transmission electron microscopy (HRTEM), relaxameter, near edge X-ray absorption fine structures (NEXAFS) and these NPs have also been applied in a performed cell study evaluating ROS production and viability. The result in this study shows that Gd implemented CeNPs are a highly promising material for theranostic applications. Schematic illustration, see Fig. 1.

## Results and Discussion

### Synthesis and Material characterization of CeNPs

CeNPs with various fractions of Gd were wet-chemically synthesized with accurate control of the Gd content. The Gd fractions were proven to be in the range of 0–50% (Gd/(Ce + Gd)) in good agreement with previous studies^33,43^. The prepared nanoparticle samples will from now on be abbreviated as CeOx (pure cerium oxide nanoparticles) or CeOx:Gd–%, where the percentage number correspond to the ICP-MS (Inductively Coupled Plasma – Mass Spectroscopy) measured ratio of Gd atoms compared to the total amount of rare-earth elements within the nanoparticles.

**Figure 2:**
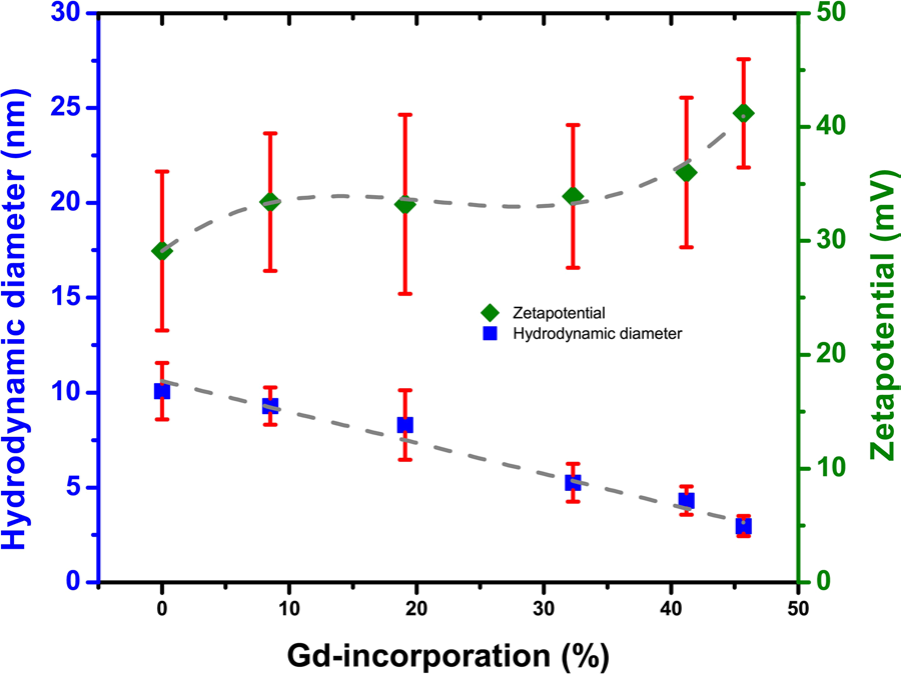
Contin number weighted hydrodynamic diameter and Zeta potential values for CeNPs with fractions of gadolinium 0%, 9%, 19%, 32%, 41% and 46% (CeOx, CeOx:Gd9%, CeOx:Gd19%, CeOx:Gd32%, CeOx:Gd41% and CeOx:Gd46%).

DLS was used for determining the hydrodynamic diameter of the as-prepared CeNPs for CeOx, CeOx:Gd9% CeOx:Gd19% CeOx:Gd32% CeOx:Gd41% and CeOx:Gd46%). The number weighted data from the contin fitting and the measured Zeta potential values of the CeOx:Gd-series are presented in Fig. 2. The hydrodynamic diameter of the CeNPs decrease linearly with increasing Gd content, and is in good agreement with earlier published results^44^. The zeta potentials increase with a sigmoidal behavior from 29.1 ± 7.0 mV for CeOx to 41.2 ± 4.8 mV for CeOx:Gd46%. 30 mV or higher indicates good colloidal stability, therefore implementation of Gd can be utilized to improve the colloidal stability in CeNPs.

**Figure 3:**
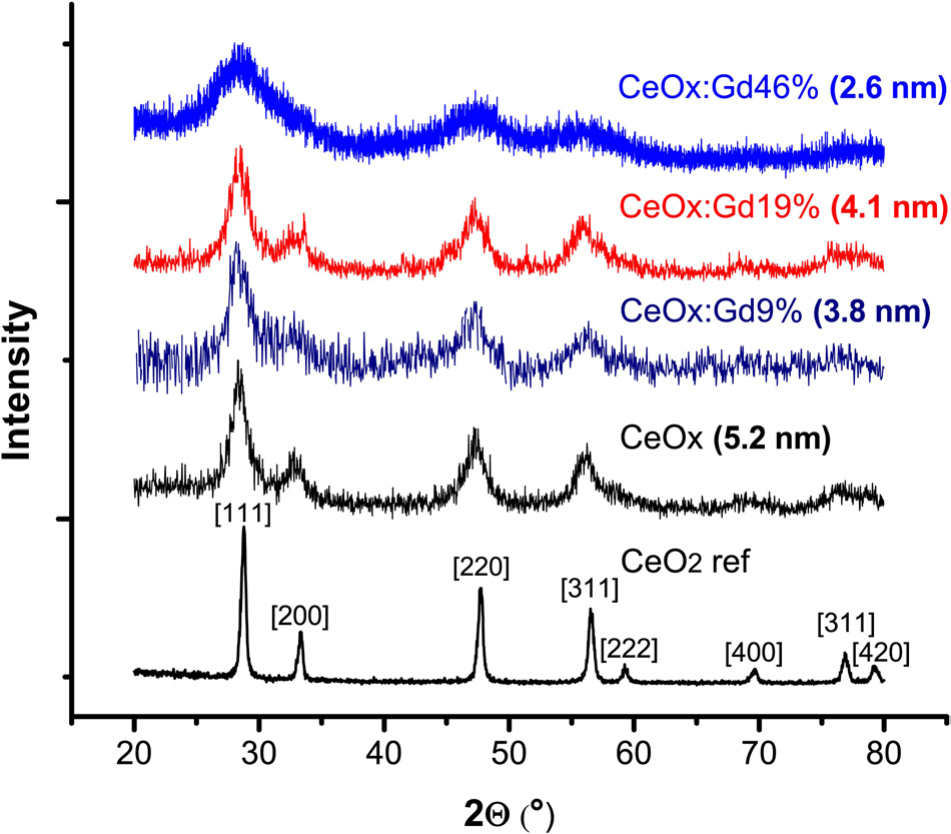
X-ray diffractograms of three CeNPs samples (CeOx, CeOx:Gd9%, CeOx:Gd19% and CeOx:Gd46%) and one reference sample of cerium oxide nanopowder (CeO_2_ ref). The calculated grain sizes of the [220] peak from the Scherrer equations are given.

The XRD patterns of the CeNPs samples CeOx, CeOx:Gd9%, CeOx:Gd19% and CeOx:Gd46% were compare to a reference sample of cerium oxide crystal in Fig. 3. All the synthesized samples exhibit the three most prominent peaks characteristic for cerium oxide, corresponding to the [111]-, [220], and [311]-planes in the cubic fluorite crystal structure^45^. Increased fraction of Gd result in broader peaks which, according to the Scherrer relationship, corresponds to a smaller particle size^46^. This observation supports the results from DLS, where a reduction in particle size upon increased Gd fraction was indicated

**Figure 4:**
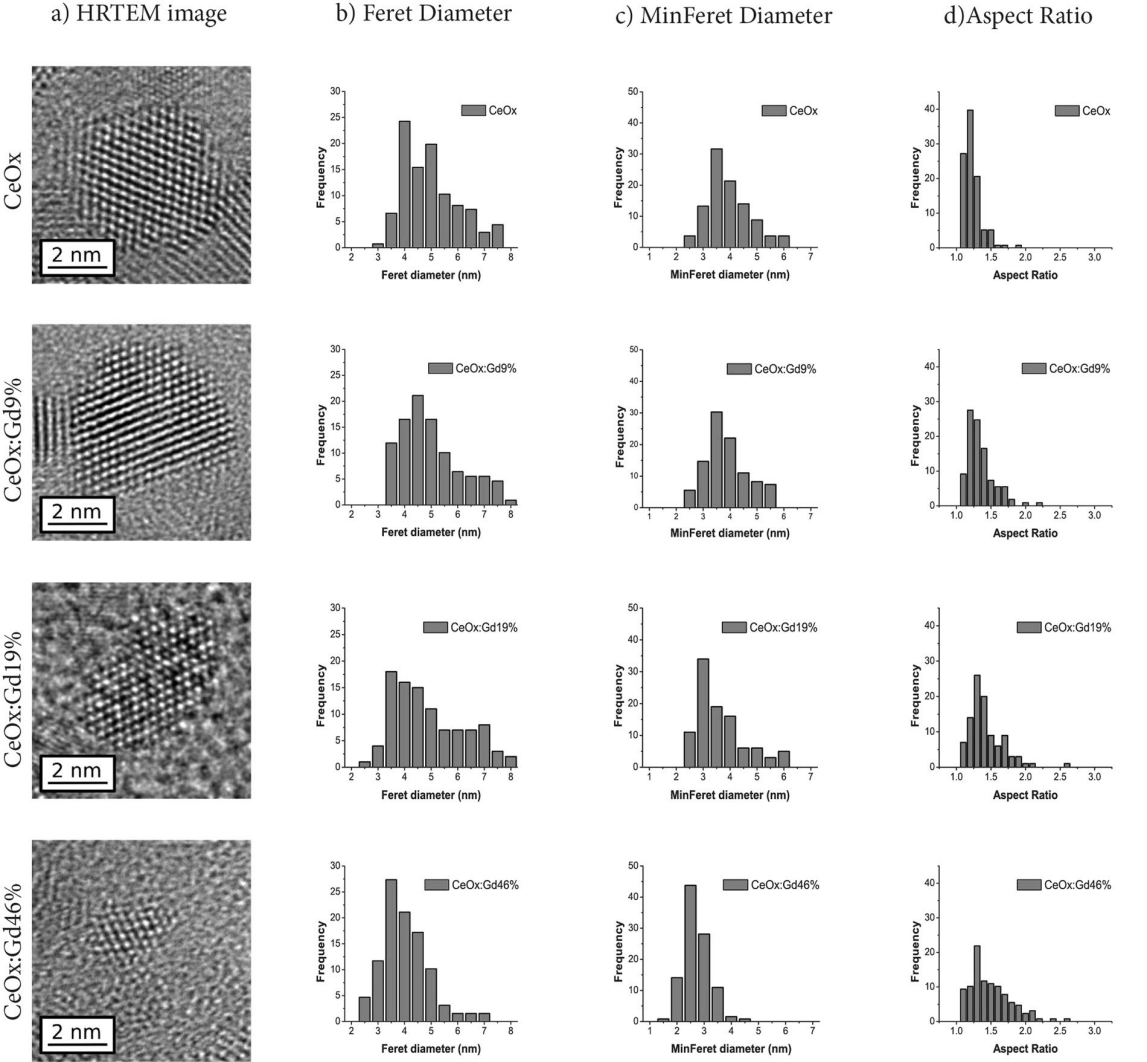
Representative HRTEM images of individual nanoparticles in (column a), distribution Feret Diameter of (column b), minimum Feret diameter (column c) and Aspect Ratio (column d) based on measurements from a minimum of 100 nanoparticles of respectively CeOx, CeOx:Gd9%, CeOx:Gd19% and CeOx:Gd46%. A full set of TEM images and size distribution for all samples (0%, 9%, 19%, 32%, 41%, 46%) are presented in supplementary material.

To confirm the particle size trend observed with both DLS and XRD, the CeNPs were analyzed by HRTEM, see Fig. 4 for CeOx, CeOx:Gd9%, CeOx:Gd19% and CeOx:Gd46%. In all cases the atomic structure of individual particles could be clearly resolved revealing that they consist of a single crystal domain, Fig. 4 (column a). Interestingly HRTEM reveals that Gd addition not only reduces the particle size but also changes its shape, from highly symmetrical cubic structures to anisotropic ovoids. This is reflected in the size distributions where there is a smaller difference between the samples Feret dimension (maximum caliper distance, Fig. 4 (column b)) compared to the minimum Feret dimension (minimum caliper distance, Fig. 4 (column c)). The Gd containing samples have a wider range of shapes and higher aspect ratio, Fig. 4 (column d) compared to the CeOx sample. Visually the lattice contrast becomes less apparent with increasing Gd content, possibly reflecting the partial disruption to the lattice structure.

### Ability Characterization; Relaxivity and Oxidation States

**Figure 5:**
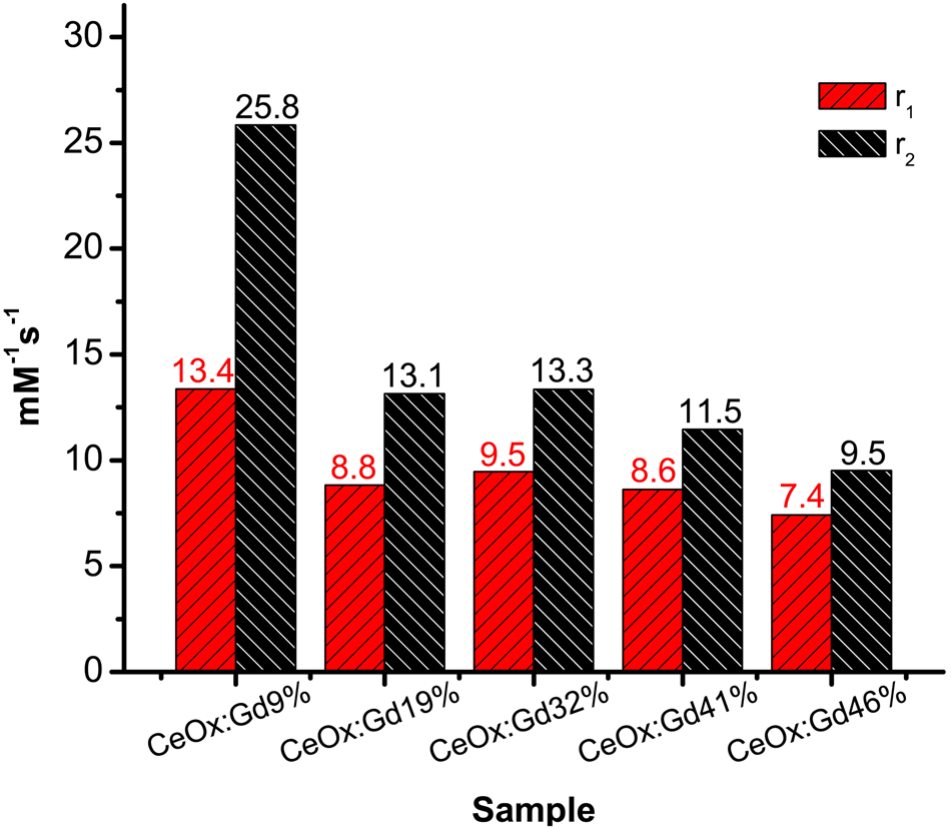
Relaxivities(r_1_ and r_2_) of CeOx:Gd:9%, CeOx:Gd:19%, CeOx:Gd32%, CeOx:Gd41% and CeOx:Gd46% are shown in the staple diagram Relaxivity plotted as a function of Gd (mM) for all five samples (9%, 19%, 32%, 41% and 46%) are presented in Supplementary material.

Relaxivity is a measure of a material’s intrinsic ability to respectively provide positive and negative contrast in for example MRI scans. Relaxivities (r_1_ and r_2_) of the Gd-containing CeNPs (Gd-CeNPs)(CeOx:Gd:9%, CeOx:Gd:19%, CeOx:Gd32%, CeOx:Gd41% and CeOx:Gd46%) are presented in Fig. 5. The R1 and R2 relaxation values are presented as a function of Gd concentration in Figure S8–S12. CeNPs with increased Gd-content display lower r_2_/r_1_ ratio and lower relaxivities per Gd. CeOx:Gd9% display the highest r_1_ and r_2_ values per Gd, indicating that it is the most efficient sample to provide contrast for MRI. The r_1_ value is typically related to positive contrast, and the CeOx:Gd9% r_1_ value at 13.4 mM^−1^ s^−1^ is about 3 times higher than the r_1_ value for Gd-based contrast agents in clinical use. Note that earlier published relaxivities on pure sub 5 nm Gd_2_O_3_ nanoparticles were found to be r_1_ = 6.9 mM^−1^ s^−1^ and r_2_ = 7.9 mM^−1^ s^−1^ ^38^, All Gd-CeNPs have r_2_/r_1_ ratio below 2 and can be considered as positive contrast enhancers^31^.

The relaxivity properties of a contrast agent depend on several parameters such as Gd to water distance, hydration number, water exchange and rotational diffusion^32^. These parameters can be affected by changes in nanoparticle shape and size. Further investigations are required for a complete understanding on how the infusion of Gd affects the relaxivity properties.

**Figure 6:**
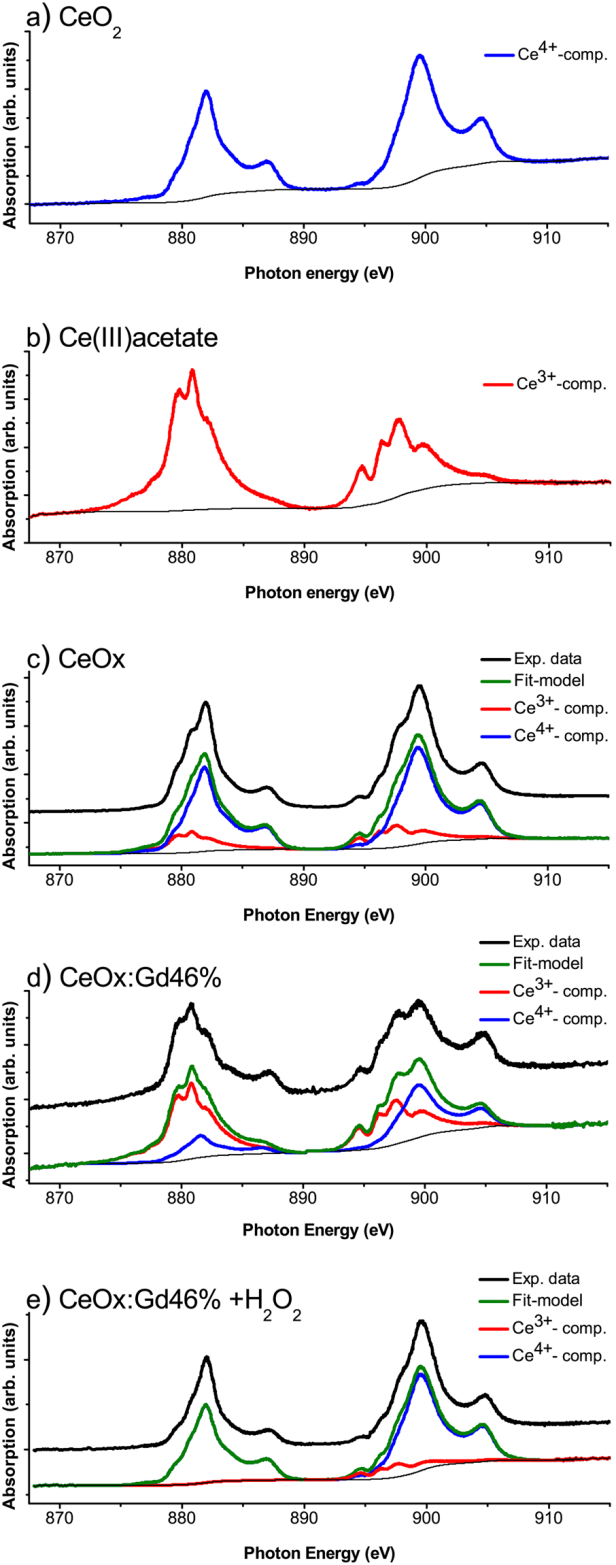
NEXAFS spectrum of (**a**) CeO_2_ nanopowder (reference), (**b**) Ce(III)acetate (reference), (**c**) CeOx, (**d**) CeOx:Gd46% and (**e**) CeOx:Gd46% treated with 10 mM hydrogen peroxide. The spectrum component analysis was performed using CasaXPS.

The antioxidant abilities of CeNPs are a direct result of the ability for Ce to switch between oxidation states 4+ and 3+ (Ce^4+^ and Ce^3+^). NEXAFS has earlier been described as a useful technique for quantifying the oxidation states of Ce using spectral component analysis^47^. In this work, NEXAFS has been used to analyses the Ce M-edge in order to investigate the oxidation states of Ce. In Fig. 6 the Ce M_4,5_ edges show a multiplet splitting structure, reflecting transitions of 3d core electrons to unoccupied states of p- and f-like symmetries^48^. The characteristic features of Ce M-edge has formerly been described by Magnuson *et al*.^49^ and in Fig. 6a and b the Ce^3+^ and Ce^4+^ oxidative states are presented. The M5 and M4 peaks vary in both intensity and shape depending on the oxidation state of the materials. Previous attempts to understand NEXAFS spectra of cerium oxide have been done in the framework of theoretical schemes involving model Hamiltonians. Gunnarsson and Schönhammer^50^ used the Anderson impurity model. Kucheyev *et al*.^51^ estimated atomic multiplets for the M4,5 edge of Ce^4+^ and Ce^3+^. However, to the best of our knowledge, first principle calculations, perhaps the most reliable approach for understanding of the electronic structure of a material, have not been reported for the M-edge of cerium oxide. With this in mind, we carried out *ab-initio* DFT calculations of NEXAFS M4,5 spectra of CeO_2_ and Ce_2_O_3_ in the framework of density functional theory^52^.

**Figure 7:**
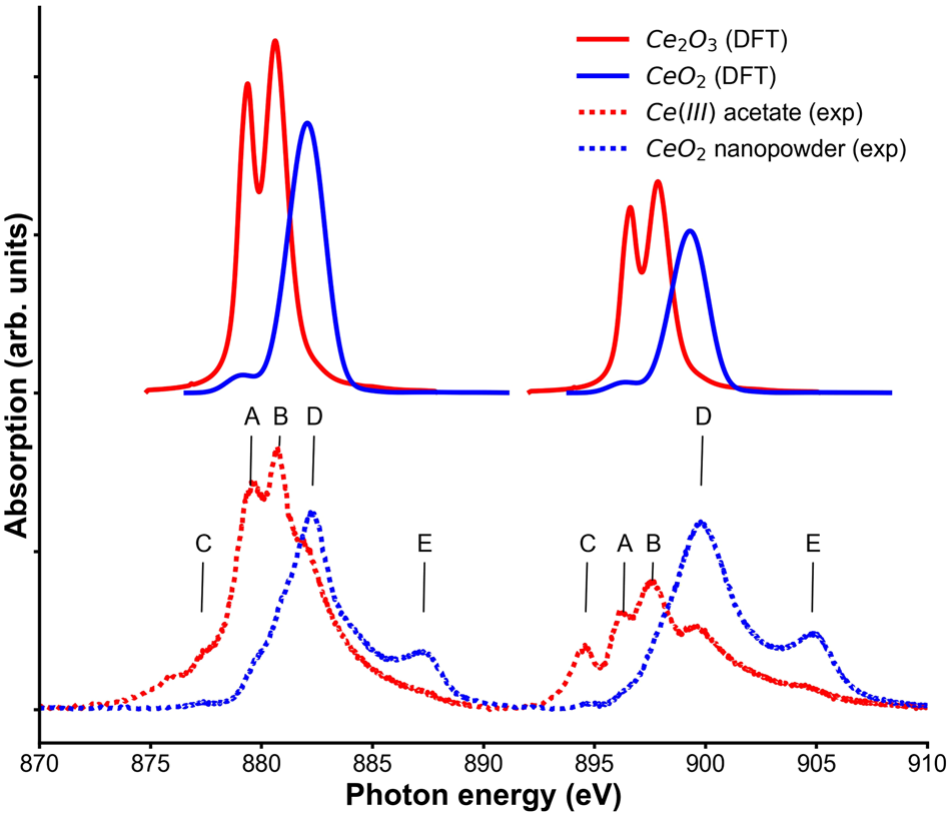
Calculated NEXAFS spectra for CeO_2_ and Ce_2_O_3_. Both spectra are shifted to experimental values.

The employed methodology allows us to account for a core-hole explicitly, by removing one electron from 3d-states. The calculations were performed for bulk CeO_2_ in fluorite structure and hexagonal Ce_2_O_3_. The results are shown in Fig. 7, and compared to the performed experiment. The absolute absorption energies have been shifted for direct comparison with experimental spectra. It has been found that upon transition from Ce^4+^ to Ce^3+^ the peak shifts by 2 eV toward smaller energies, which is in a good agreement with experimental spectra. In Ce_2_O_3_ the main peak has a doublet structure consisting of A and B features, which is reproduced in the calculations. The origin of this structure can be explained from the density of 4 f states (see Figure S14 in Supplementary material) splitting between spin-up and spin-down.

In Fig. 7 Ce_2_O_3_ spectra display a prominent feature C. This absorption peak lies in the band gap, which means that it corresponds to an excitation that cannot usually be reproduced in independent particles approximations such as DFT. The CeO_2_ M-edge has a very prominent satellite E which corresponds to charge transfer from O2p to Ce4f state. Again, this type of excitation is usually not reproduced by DFT and a different methodology would be required, e.g. based on the configuration interaction method^53^.

Thus, the provided allow us to identify all the spectral features corresponding to different oxidation states and understanding of the origin of all components of the spectra suggests that the spectra of cerium oxide nanoparticles is a combination of M-edge of atoms with different oxidation states Ce^3+^ and Ce^4+^ and no size specific features are observed, justifying the component analysis presented below.

In the component analysis, Baltrusaitis *et al*.’s procedure^54^ was employed to facilitate the spectral analysis of the complex multiplet structure of cerium oxide’s M5 and M4 peak. Considering the origin of the spectral features obtained in DFT calculations, the reference spectra of the different oxidations states respectively were utilized as spectral components to track the respectively oxidation states in the CeNPs, Fig. 6c and d. The oxidation state 3^+^ increases with the Gd fraction, due to both implementation of Gd^44^ and the reduction in size^25^. The shift in oxidation states of CeOx:Gd46% in presence of the ROS hydrogen peroxide is demonstrated in Fig. 6c and d. In absence of hydrogen peroxide a contribution from both Ce^3+^ and Ce^4+^ were obtained, see Fig. 6d while in presence of 10 mM hydrogen peroxide the Ce^3+^-component is dramatically reduced Fig. 6e. The ability to switch oxidation state in presence of ROS is a strong indication that the particles behave as an active antioxidant material.

### Biocompatibility and Antioxidant Assay

The ROS-scavenging properties of CeNPs were evaluated in an *in vitro* model, utilizing dichloro-dihydro-fluorescein (DCF-DA), a commonly used fluorescent probe for quantifying ROS^55,56^, human neutrophils and phorbol myristate acetate (PMA) for inducing neutrophil ROS-production^57^. Neutrophils show the most potent production of ROS among the phagocytic leukocytes and produce large amounts of ROS via the NADPH-oxidase complex in a process known as “the respiratory burst”. PMA is a strong inducer of NADPH-oxidase mediated ROS^58^. In other types of cells mitochondrial production is the source of ROS. For example, some tumor cells are shown to have elevated levels of mitochondria derived ROS. In these cells ROS function as a signaling molecule influencing for example cell proliferation^59^.

**Figure 8:**
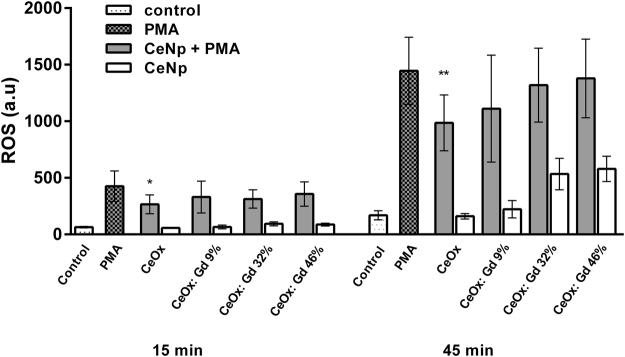
Neutrophil granulocytes were isolated from human whole blood and incubated with CeNPs in presence or absence of PMA (1 µM). The CeNPs concentration were set to 50 µg/ml of cerium for all nanoparticle samples. ROS production was measured using DCF-DA for 60 minutes. N = 4–8, minimum duplicate samples. ROS production (a.u) ± S.E.M. *P ≤ 0.05, **P ≤ 0.01 (paired t-test, GraphPad Prism ver. 6.07).

In the present paper possible scavenging properties of CeNPs were assessed in the robust model with neutrophils described above. As these cells produce large amounts of ROS, scavenging effects in this cellular system would indicate a great potential. ROS-production was traced for 1 h and the values acquired at 15 min and 45 min, Fig. 8. All prepared CeNPs display a modulatory effect on neutrophil production of ROS compared to the PMA-reference, the CeOx sample displayed significant ROS scavenging properties compared to the control. In control experiments without addition of PMA, CeOx does not induce any ROS production (white bars Fig. 8). For NPs with higher fractions of Gd, ROS production is detected after 45 minutes of incubation. This is in good agreement with a previous paper^60^, where we showed that non-coated gadolinium oxide nanoparticles induce ROS production from neutrophils, which is why they require a biocompatible coating to minimize ROS induction.

In addition to the ROS experiments, biocompatibility was assessed in neutrophils (short term- up to 3 hours) as well as in human fibroblasts (long term, 7-day assay) using resazurin and crystal violet-based assays, respectively. The results are shown in Figures S15 and S16 Supplementary material. In neutrophils, no decrease of viability was observed for any of the tested nanoparticles. Reduced neutrophil metabolic activity, by means of NP induced toxicity, could be excluded. This strongly indicates that the inhibitory effects of Ce based NPs on ROS production is due to their scavenger properties. In addition, no toxicity was observed in the fibroblasts during a long term (7 days) assay. Interestingly, fibroblasts instead showed a proliferative response following treatment with some of the CeNPs. In summary, CeNPs is a highly promising material for its biocompatibility and antioxidant properties.

## Conclusion

Cerium oxide nanoparticles (CeNPs) have been designed through wet-chemistry with controlled infusion of gadolinium (Gd), enabling ROS-scavenging and MRI contrast enhancement properties into one theranostic nanoprobe. Increasing Gd content reduces the size and increases the aspect ratio of the produced particles. The diagnostic MRI contrast enhancement properties of designed Gd-CeNPs display superior r_1_-relaxivities, 7–13 mM^−1^ s^−1^ compared to Gd-based contrast enhancers in use at the clinic. The NEXAFS results show 1) increase of Ce^3+^-content in CeNPs upon Gd implementation, thereby tuning the ROS-scavenging properties and 2) clear shift from oxidation state 3+ to 4+ upon H_2_O_2_-treatment, indicating antioxidant-behavior. The origin of the most prominent features in the Ce3d NEXAFS spectra of oxidation state 3+ and 4+, could be explained with *ab initio* calculations.

Pure CeNPs display significant ROS-scavenging properties in samples with PMA-activated human neutrophils. Clearly, Gd-CeNPs with low Gd content, i.e. CeOx:Gd9%, indicate ability to scavenge ROS upon PMA-activation, which indicates that incorporation of Gd in CeNPs is a successful strategy to reduce harmful effects from Gd, which can be further minimized with biocompatible coating and functionalization.

The aim was to combine the therapeutic properties of Ce together with the diagnostic properties of Gd into one single probe, to obtain an agent with potential for theranostics. Gd-CeNPs are successfully customized in this work with elemental composition controlled by fine-tuning the relative ratio of Ce and Gd. The theranostic capability is nicely demonstrated by the CeOx:Gd 9% sample, which displays 1) the superior r_1_-value 13.4 mM^−1^ s^−1^, 2) clear crystal structure, 3) clear ROS scavenging capability and 4) well-tolerated by both neutrophils and fibroblasts.

This study is limited to *in vitro* experiments, and to confirm the theragnostic properties of the CeNPs also in a more biologically complex setup *in vivo* studies should be done in the future. Further studies on mechanistic principles and pathways are under way.

All the above considered, the results in this study clearly indicate that Gd-CeNPs have potental for future biomedical applications and are especially interesting for development of theranostic agents.

## Material and Methods

### Synthesis and Purification of Nanoparticles

Cerium oxide nanoparticles have been prepared with 0–50 mol% gadolinium content by using a simple wet-chemical synthesis at room-temperature. All solutions used in the synthesis were pumped and purged with nitrogen gas. First 0.5 mmol of Cerium(III)- and Gadolinium(III)-acetate were dissolved in 5.48 ml of a 50/50 MilliQ-water and Triethylene Glycol (TEG) solution. 0.52 ml of 50/50 TEG and 30% ammonium hydroxide added dropwise to the solution under constant stirring. Thereafter continued the stirring and the reaction for two hours before the synthesis was stopped.

The nanoparticle solutions were centrifugated at 3000 g for 5 min and the supernatants were removed before the nanoparticles were dispersed in MilliQ-water. Thereafter were the nanoparticles dialysed (Slide-A Lyzer® MINI Dialysis Devices, 10 K MWCO, 2 ml) against MilliQ-water at a minimum ratio of 1:1000 for 24 hours with 2 water exchanges. After dialysis the nanoparticle solutions were filtered using Acrodisc® 25 mm syringe filter with w/0.1 µm Supor® membrane.

### Instrumentation

#### Inductively Coupled Plasma Mass Spectroscopy

The ICP-MS measurements were performed by ALS Scandinavia AB.

#### Relaxation

The relaxation or relaxivity studies were carried out with a Bruker minispec mq60 NMR analyzer at 40 °C using a magnetic field of 1.41 T. MilliQ water was used for diluting the nanoparticle samples and each sample were temperature stabilized for 4 min before measurement.

#### Dynamic Light Scattering

Dynamic Light Scattering measurements were performed on an ALV/DLS/SLS-5022F system from ALV-GmbH, Langen Germany, using a HeNe laser at 632.8 nm operating at 20 °C and measuring at 90° scattering angle. The samples were thermally stabilized in a thermostat bath at 20 °C for 15 min before measurement.

The cumulant analysis presented polydispersity index about 0.3–0.4 for the CeOx:Gd samples, concluding presence of larger aggregates. Therefore, contin analysis model were utilized to fit the correlation curve.

#### Transmission Electron Microscopy

Prior to analysis nanoparticles were deposited directly onto to an amorphous carbon film supported on a copper TEM grid. All measurements were taken with a FEI Tecnai G2 (FEI) operated at 200 kV. For particle size measurements, individual particles where identified manually from their lattice fringes after which the Feret and minimum Feret dimensions where determined using automatic image analysis (ImageJ) and the resulting size distributions were fitted with a log-normal distribution.

#### Zeta Potential

Zeta Potential measurement were performed on a Malvern Zetasizer Nano ZS90 operated at 25 °C using DTS1070 cuvettes.

#### X-ray Diffraction

Powder of CeNPs samples for XRD were obtained in two different ways: 1) After synthesis the solutions were centrifugated and the supernatant was removed, and the pellets were dispersed in ethanol. This washing procedure were repeated and thereafter the sample solution was dried using rotary evaporator to obtain powder of the nanoparticles. 2) After dialysis, the samples were frozen in liquid nitrogen and freeze-dried.

The powder was examined with a Phillips XRD diffractometer using Cu Kα radiation (λ = 1.5418 Å, 40 kV, 40 mA). The 2θ-step size was 0.030° and the time per step were at least 3.75 seconds.

#### Near Edge X-ray Absorption Fine Structures

The samples for NEXAFS were prepared by dropping 15–20 µl of respectively nanoparticle solution at gold surfaces and dried with aid of N_2_-gas. The measurements were carried out at beamline D1011 at the synchrotron storage ring MAX II at MAX-Lab I Lund, Sweden. Retardation voltage was set to −700 V for Ce3d.

#### Computational modelling

Density functional theory (DFT) calculations were performed in the all-electron full-potential augmented plane wave + local orbital (APW + lo) scheme using the WIEN2K^52^ software package, with the local density approximation (LDA) for exchange-correlation functional. Hubbard correction^61^ U of 6 eV have been applied to f-states. CeO2 in fluorite structure with 96 atoms in a supercell and Ce_2_O_3_ in hexagonal structure with 60 atoms in a supercell have been calculated. The calculations were converged self-consistently utilizing a 2 × 2 × 2 k-mesh, including spin-orbit coupling and spin polarization. A denser k-mesh was used for plotting the spectra.

#### Isolation of human neutrophil granulocytes and measurement of reactive oxygen species

Blood samples were collected from healthy, non-medicated volunteers at the Blood Bank at Linköping University Hospital, Sweden, in accordance with the criteria for ethical approval in humans (2003:460). Neutrophil granulocytes were isolated from heparinized human whole blood drawn. In short, neutrophils were separated using polymorphprep (Axis Shield PoC AS, Oslo, Norway) and centrifugation (480 × g for 40 min at r.t.) according to protocols previously described^62,63^. Separated cells were washed (2×, 480 × g for 10 min) in phosphate buffered saline (PBS; 0.01 M phosphate buffer, 0.0027 M potassium chloride and 0.137 M sodium chloride, pH 7.4) and any remaining erythrocytes were removed by brief hypotonic treatment. Isolated neutrophils were resuspended in HEPES buffer (145 mM NaCl, 5 mM KCl, 1 mM MgSO4, 10 mM HEPES, 10 mM Glucose, pH 7.4) and cell concentration was adjusted to 2 × 10^6^/mL using a Bürkner chamber. Measurement of neutrophil production of reactive oxygen species was performed using 2, 7-dichlorofluorescein diacetate (DCF-DA, Sigma Aldrich, St. Louis, MO, USA). Briefly, cells were incubated with DCF-DA (2 µm) at r.t for 20 min. Neutrophils were then exposed to cerium oxide nanoparticles in 96 well plates at 37 degrees C. Nanoparticle concentration were normalized to 50 µg/ml cerium oxide. DCF fluorescence was followed in a plate reader (model, company), excitation 485 ± 10 nm, emission 530 ± 20 nm during 60 minutes. Phorbol 12-myristate 13-acetate (PMA; Sigma) was used as positive control. All samples were run in at least duplicates and in up to 8 different blood donors.

Viability was assessed using PrestoBlue cell viability reagent (Thermo Scientific) according to the manufacturer’s instructions. Samples were run in triplicates and performed using 3 different blood donors.

### Toxicity assay in human fibroblasts

Human foreskin fibroblasts (AG01518; passages 12–24; Coriell Institute for Medical Research, Camden, NJ) were cultured in Dulbeccco’s modified Eagle’s Medium (DMEM) supplemented with 2 mM glutamine, 1% non-essential amino acids, 100 IU/mL penicillin, 50 μg/mL streptomycin, and 10% fetal bovine serum (all from GIBCO, Paisly, UK). Upon confluence cells were detached by trypsinization (0,25% trypsin and 0,02% EDTA) and seeded in 12 well plates at a density of 10000 cells/well, left to attach for 24 h followed by treatment with NPs ([Ce] = 50 µg/mL). At day 7 cytotoxic effects were evaluated by crystal violet staining. Briefly, cells were fixed in 4% paraformaldehyde for 20 min, stained with crystal violet (0,04% in 1% ethanol) for 20 min before being washed and air dried. Solubilization (1% SDS) was done prior to recording of absorbance at 550 nm in a Victor plate reader (EG & G Wallac, Upplands Väsby, Sweden)^64^.

## Electronic supplementary material

Supplementary information
