# Supplementary material for: Cerium oxide nanoparticles with antioxidant capabilities and gadolinium integration for MRI contrast enhancement
Source: Sci Rep. 2018 May 3;8:6999. doi: 10.1038/s41598-018-25390-z (PMC5934375; doi:10.1038/s41598-018-25390-z)
Supplement: Supplementary file 1 — Supplementary information [file 41598_2018_25390_MOESM1_ESM.docx]

# Supplementary Information

Cerium oxide nanoparticles with antioxidant capabilities and gadolinium integration for MRI contrast enhancement

Peter Eriksson^1^, Alexey A. Tal^2,5^, Andreas Skallberg^1^, Caroline Brommesson^1^, Zhangjun Hu^1^, Robert D. Boyd^3^, Weine Olovsson^2^, Neal Fairley^4^, Igor A. Abrikosov^2,5^, Xuanjun Zhang^6^ and Kajsa Uvdal^1^

^1^ Division of Molecular Surface Physics and Nanoscience, Department of Physics, Chemistry and Biology (IFM), Linköping University, SE-581 83 Linköping, Sweden

^2^ Division of Theoretical Physics, Department of Physics, Chemistry and Biology (IFM), Linköping University, SE-581 83 Linköping, Sweden

^3^ Plasma Coatings Physics, Department of Physics, Chemistry and Biology (IFM), Linköping University, SE-581 83 Linköping, Sweden

^4^ Casa Software Ltd, Bay House, 5 Grosvenor Terrace, Teignmouth TQ14 8NE, United Kingdom

^5^ Materials Modeling and Development Laboratory, National University of Science and Technology “MISIS,” 119049 Moscow, Russia

^6^ Faculty of Health Sciences, University of Macau, Macau SAR, China

# High resolution transmission electron microscopy (HRTEM)

HRTEM was used for measuring the Feret, MinFeret and Aspect Ratios of at least 100 nanoparticles for each as-prepared CeNPs. The results are displayed as mean values, along with their standard deviations,in Table S1 and as histograms in Figure S1. In Figure S2-S7 a representative HR-TEM image from each sample is presented.

| Sample | Feret | Std | MinFeret | Std | Aspect Ratio | Std |
| --- | --- | --- | --- | --- | --- | --- |
| CeOx | 4,72 | 1,07 | 3,69 | 0,80 | 1,18 | 0,13 |
| CeOx:Gd9% | 4,75 | 1,19 | 3,59 | 0,76 | 1,29 | 0,20 |
| CeOx:Gd19% | 4,69 | 1,42 | 3,37 | 0,89 | 1,37 | 0,24 |
| CeOx:Gd32% | 3,90 | 1,17 | 2,63 | 0,62 | 1,39 | 0,31 |
| CeOx:Gd41% | 3,79 | 0,85 | 2,69 | 0,65 | 1,40 | 0,27 |
| CeOx:Gd46% | 3,78 | 0,88 | 2,46 | 0,46 | 1,44 | 0,29 |

**Table S 1** The mean values with standard deviation of Feret, MinFeret and Aspect Ratio for each as-prepared CeNPs.

##
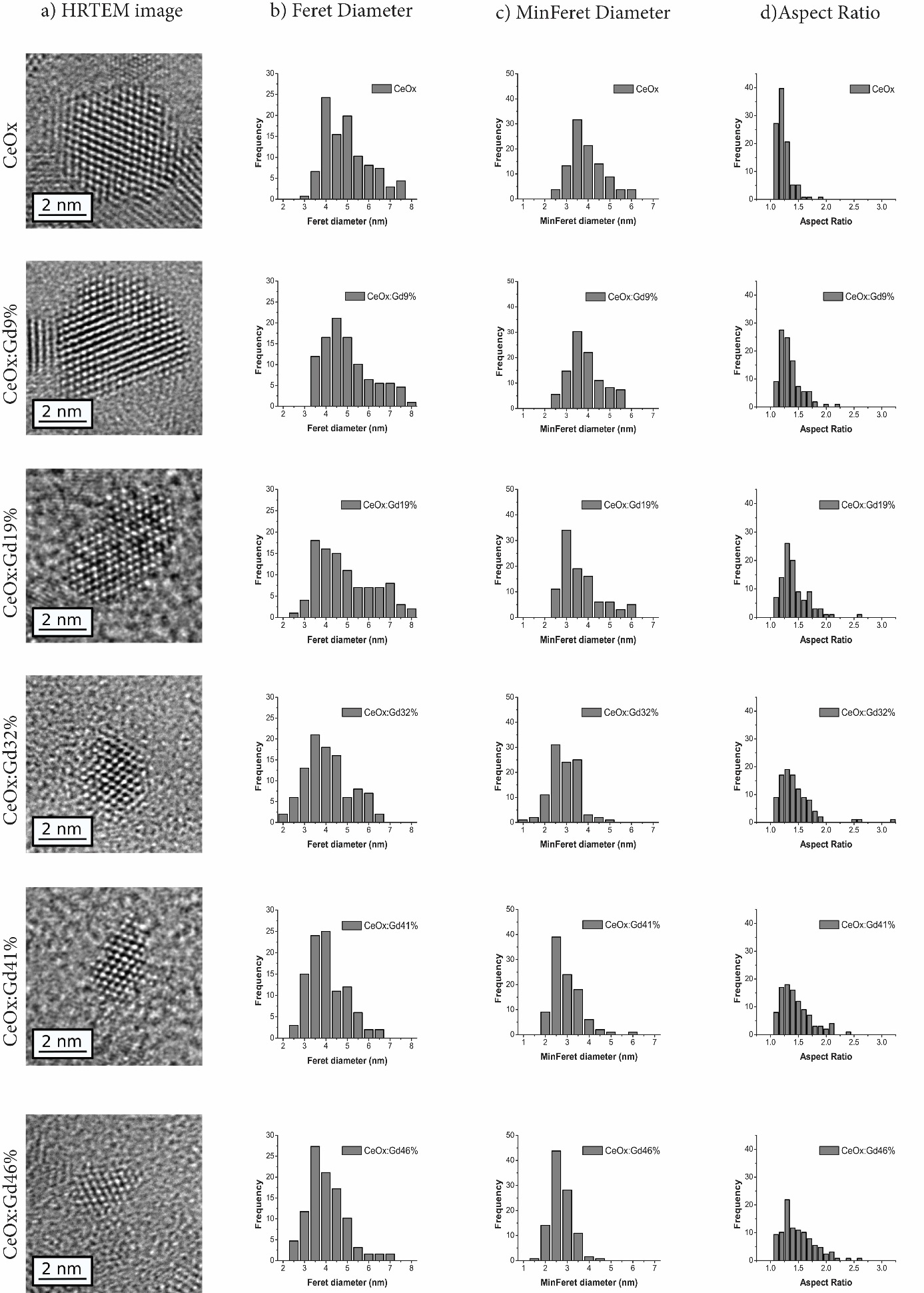


**Figure S 1** Representative HR-TEM images of individual nanoparticles in column a), distribution of Feret Diameter column b), minimum Feret diameter column c) and Aspect Ratio column d) all based on measurements from a minimum 100 nanoparticles. of respectively CeOx, CeOx:Gd9%, CeOx:Gd19%, CeOx:Gd32%, CeOx:Gd41% and CeOx:Gd46%.

## Representative HRTEM images

## CeOx





**Figure S 2** HRTEM-image of the CeOx sample

## CeOx:Gd9%


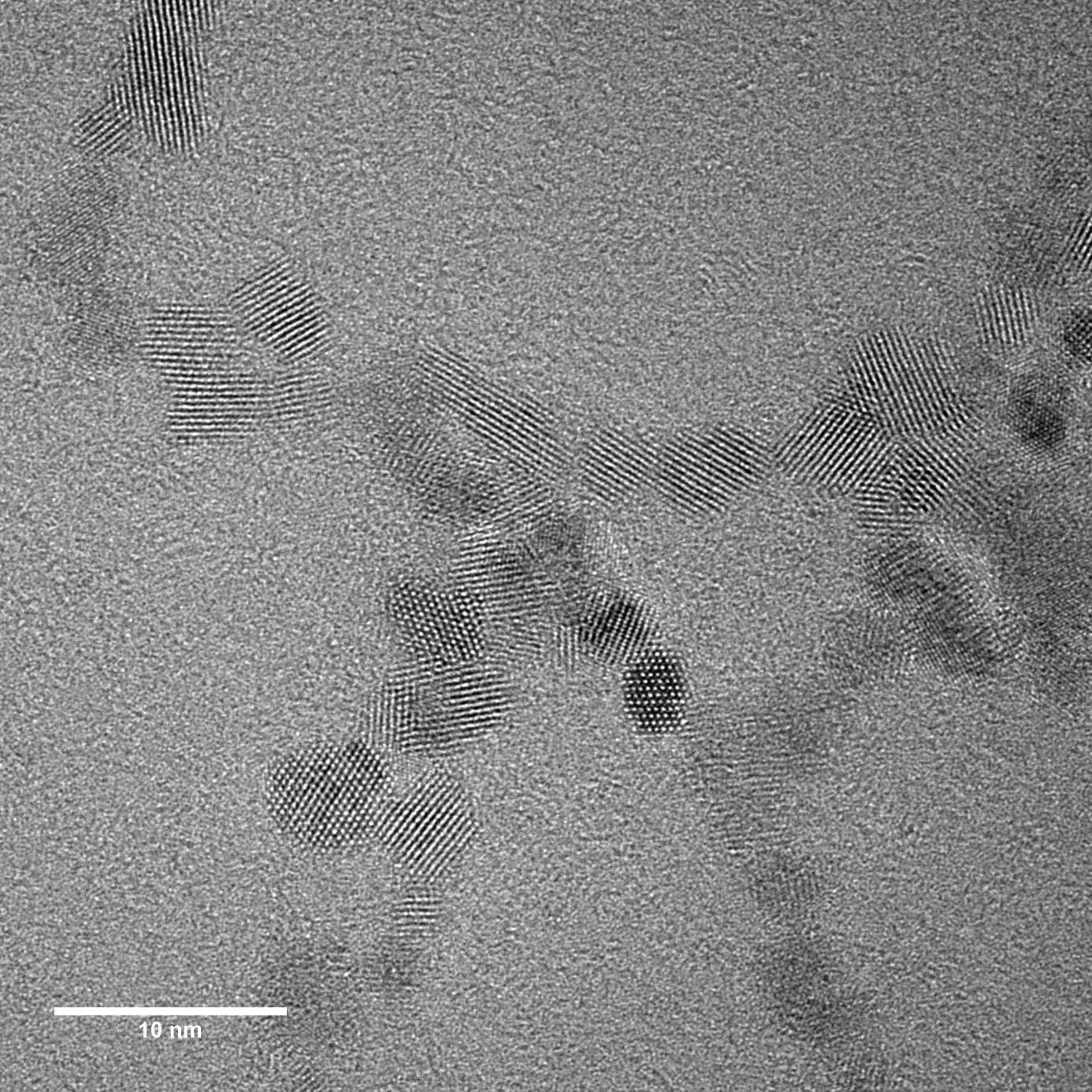


**Figure S 3** HRTEM-image of the CeOx:Gd9% sample

## CeOxGd19%


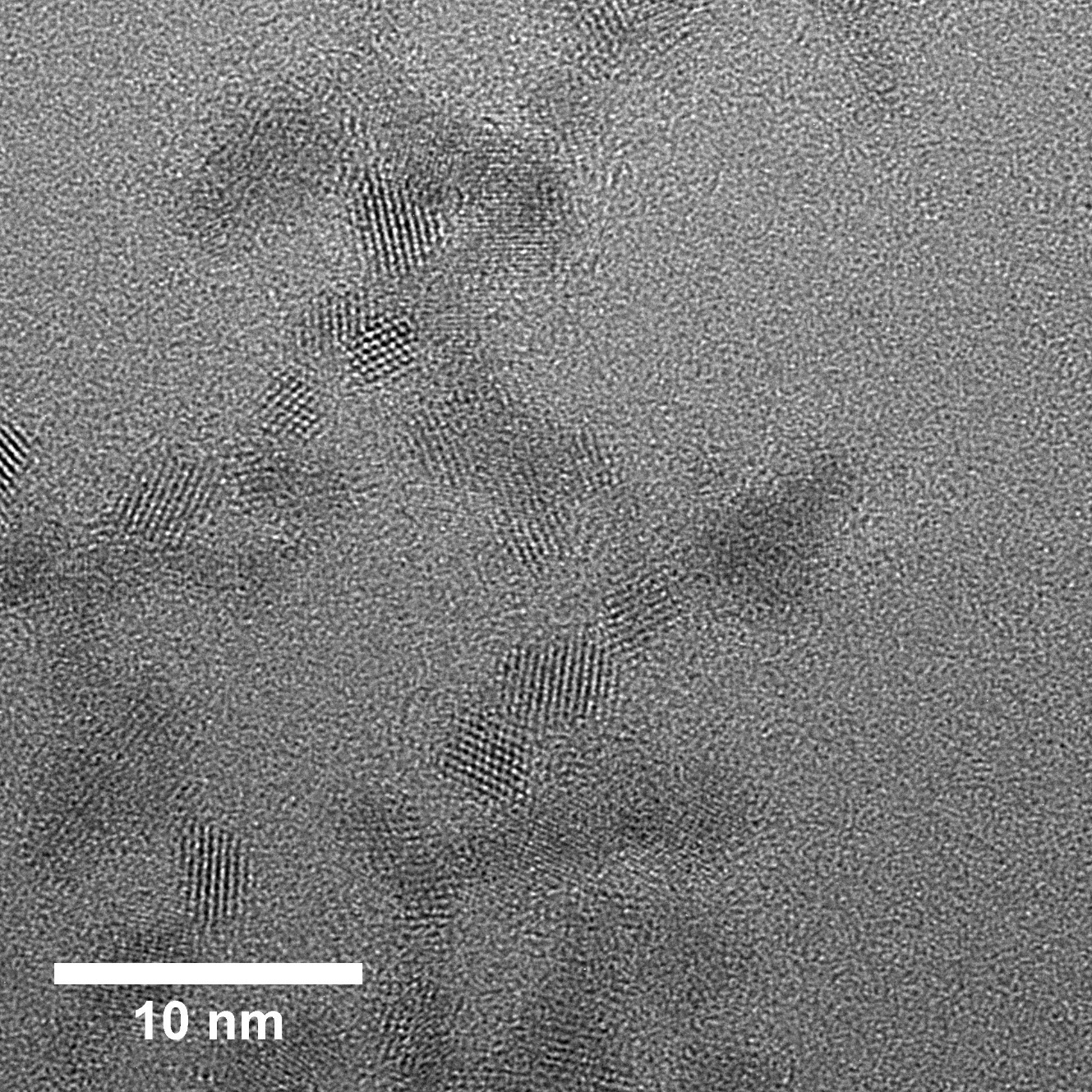


**Figure S 4** HRTEM-image of the CeOx:Gd19% sample

## CeOxGd32%





**Figure S 5** HRTEM-image of the CeOx:Gd32% sample

## CeOxGd41%


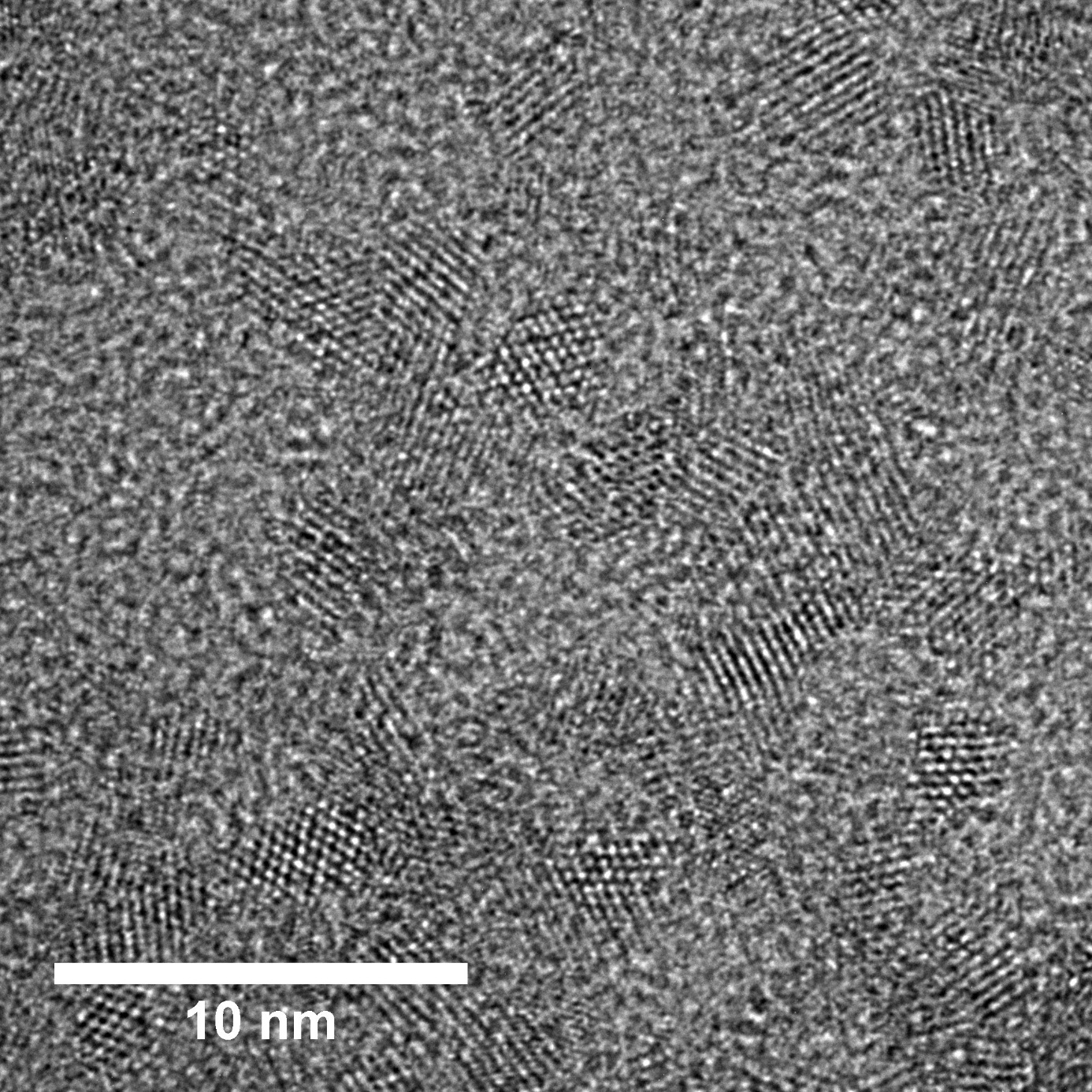


**Figure S 6** HRTEM-image of the CeOx:Gd41% sample

## CeOxGd46%





**Figure S 7** HRTEM-image of the CeOx:Gd46% sample

# Relaxvivity

The relaxation values R1 and R2 for various concentration gadolinium (Gd) have for respectively cerium oxide nanoparticles (CeNPs) sample been plotted in Figures S8-S12. The linear slopes fitting respectively the R1 and R2 values correspond to the relaxivities r_1_ and r_2_. Further discussion reflecting the relaxivities for each CeNPs sample is given in the article.

In Figure S 13, rare earth (RE)-relaxivities (RE=Ce+Gd) are presented, e.g. calculated relaxivities based on the combined cerium and gadolinium concentration. Results from the CeOx sample show that the relaxivity contribution from cerium is negligible

**Figure S 8** Fitted relaxivities r_1_ and r_2_ for CeOx:Gd9%

**Figure S 9** Fitted relaxivities r_1_ and r_2_ for CeOx:Gd19%

**Figure S 10** Fitted relaxivities r_1_ and r_2_ for CeOx:Gd32%

**Figure S 11** Fitted relaxivities r_1_ and r_2_ for CeOx:Gd41%

**Figure S 12** Fitted relaxivities r_1_ and r_2_ for CeOx:Gd46%

**Figure S 13** RE-Relaxivities (r_1_ and r_2_) of CeOx, CeOx:Gd9%, CeOx:Gd19%, CeOx:Gd32%, CeOx:Gd41% and CeOx:Gd46% are shown in staple diagram. Note that the relaxivities are calculated against rare earth concentration.

# Analysis of NEXAFS Ce M4,5

In order to explain the fine structure of M4,5 NEXAFS the density of 4f states have been analysed. As shown in Figure S14, the splitting between spin-up and spin-down results in a doublet structure. While in CeO_2_ no spin-polarization is present and only a singlet peak appears. The hybridisation between 5d and 4f sates is relatively small for Ce_2_O_3_ and does not show any major effect on the spectra. For the case of CeO_2_ no hybridization is observed.


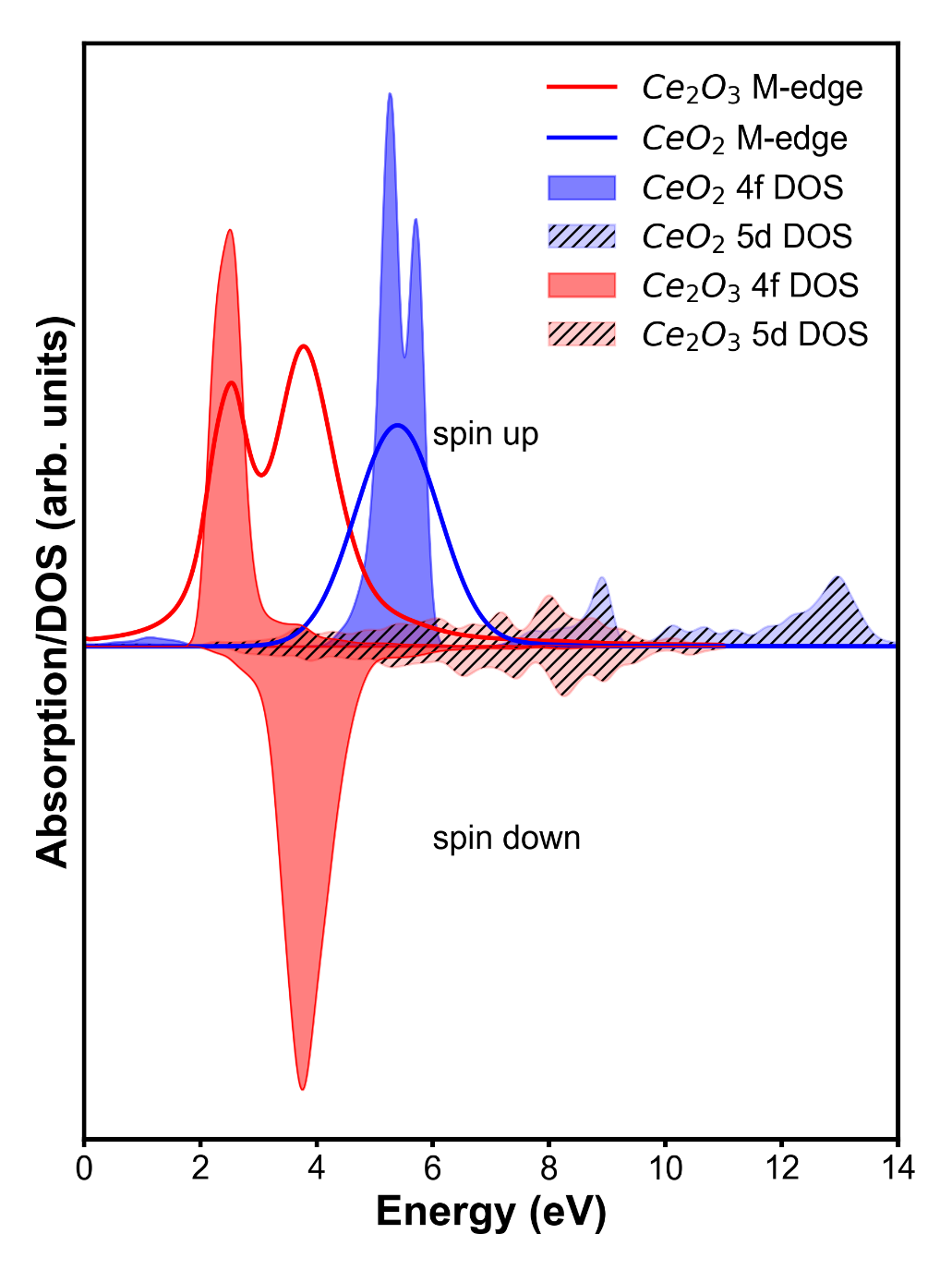


**Figure S 14** Calculated electronic density of states (DOS, shaded) of 4f and 5d states and NEXAFS M4,5 (solid lines) for CeO_2_ (blue) and Ce_2_O_3_ (red)

# Viability Assay

Viability of human neutophils treated with CeNPs were measured using a reazurin based assay. The assay have previously been used for measuring viability on human neutrophils (Leukoc. Biol. 100: 791–799; 2016.). Human neutrophils were obtained from three different blood donors. The results in Figure S15 clearly show that the neutrophils for all samples show same level of metabolic activity as for untreated control cells (reference sample normalized to 100% viability). Results from long term assay using human fibroblasts confirmed stable system without any recordable toxicity.





**Figure S 15** Neutrophils (1 * 10 ^6^ mL) were treated with NPs ([Ce ]= 50 µg/mL) and metabolic activity was analyzed using a resazurin based assay performed according to the manufacturer’s instructions


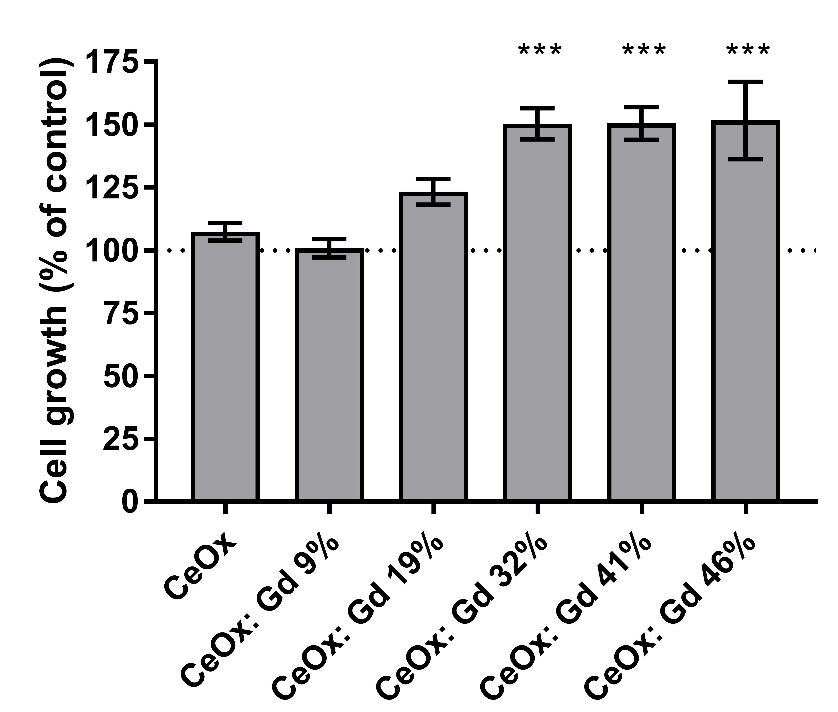


**Figure S 16** Human fibroblasts were seeded in 12-well plates (10000 cells/well, triplicates) and after 24 h treated with NPs ([Ce ]= 50 µg/mL) . Cytotoxic effects were evaluated at day 7 using crystal violet staining. Results are presented as % growth (± S.E.M) compared to untreated control. * = P ≤ 0.05, ** = P ≤ 0.01 *** = P ≤ 0.001 (one-way ANOVA followed by Bonferroni’s multiple comparison test, GraphPad Prism ver. 6.07)
